# Dr. Ira Van Gieson

**Published:** 1913-05

**Authors:** 


Dr. Ira Van Gieson of New York died Mar. 24 from interstitial nephritis, after an illness of several weeks at the age of 47 years. He was born in New York and received his medical degree from the College of Physicians and Surgeons in 1885. He was for a time instructor at the College and later was director of the Pathological Institute. He was well known as a pathologist, and at the time of his death was connected with the research laboratory of the Board of Health. He was the son of Dr. Ransford E. Van Gieson of Brooklyn, who survives him.

